# A retrospective multicentric observational study of trastuzumab emtansine in HER2 positive metastatic breast cancer: a real-world experience

**DOI:** 10.18632/oncotarget.18176

**Published:** 2017-05-25

**Authors:** Patrizia Vici, Laura Pizzuti, Andrea Michelotti, Isabella Sperduti, Clara Natoli, Lucia Mentuccia, Luigi Di Lauro, Domenico Sergi, Paolo Marchetti, Daniele Santini, Emanuela Magnolfi, Laura Iezzi, Luca Moscetti, Agnese Fabbri, Alessandra Cassano, Antonino Grassadonia, Claudia Omarini, Federico Piacentini, Andrea Botticelli, Ilaria Bertolini, Angelo Fedele Scinto, Germano Zampa, Maria Mauri, Loretta D’Onofrio, Valentina Sini, Maddalena Barba, Marcello Maugeri-Saccà, Ernesto Rossi, Elisabetta Landucci, Silverio Tomao, Antonio Maria Alberti, Francesco Giotta, Corrado Ficorella, Vincenzo Adamo, Antonio Russo, Vito Lorusso, Katia Cannita, Sandro Barni, Lucio Laudadio, Filippo Greco, Ornella Garrone, Marina Della Giulia, Paolo Marolla, Giuseppe Sanguineti, Barbara Di Cocco, Gennaro Ciliberto, Ruggero De Maria, Teresa Gamucci

**Affiliations:** ^1^ Division of Medical Oncology 2, IRCCS Regina Elena National Cancer Institute, Rome, Italy; ^2^ UO Oncologia Medica I, Ospedale S. Chiara, Dipartimento di oncologia, dei trapianti e delle nuove tecnologie, Azienda Ospedaliera Universitaria Pisana, Pisa, Italy; ^3^ Bio-Statistics Unit, IRCCS Regina Elena National Cancer Institute, Rome, Italy; ^4^ Department of Medical, Oral and Biotechnological Sciences, Centro Scienze dell’Invecchiamento e Medicina Traslazionale (CeSI-MeT), Chieti, Italy; ^5^ Medical Oncology Unit, ASL Frosinone, Frosinone, Italy; ^6^ Medical Oncology Unit, Policlinico Sant'Andrea, Rome, Italy; ^7^ Medical Oncology, Policlinico Universitario Campus Bio-Medico, Rome, Italy; ^8^ Division of Medical Oncology, Department of Oncology and Hematology, University Hospital of Modena, Modena, Italy; ^9^ Division of Oncology, Complesso Ospedaliero Belcolle, AUSL Viterbo, Viterbo, Italy; ^10^ Department of Medical Oncology, Catholic University of Sacred Heart, Rome, Italy; ^11^ Division of Medical Oncology, Department of Medical and Surgical Sciences for Children & Adults, University Hospital of Modena, Modena, Italy; ^12^ Medical Oncology, Ospedale San Giovanni Calibita Fatebenefratelli, Rome, Italy; ^13^ Oncology Unit, Nuovo Regina Margherita Hospital, Rome, Italy; ^14^ Division of Oncology, San Giovanni Hospital, Rome, Italy; ^15^ Department of Medico-Surgical Sciences and Biotechnologies, “Sapienza” University of Rome, Oncology Unit, Istituto Chirurgico Ortopedico Traumatologico, Latina, Italy; ^16^ Medical Oncology, Sandro Pertini Hospital, Rome, Italy; ^17^ Division of Medical Oncology, IRCCS, Giovanni Paolo II Hospital, Bari, Italy; ^18^ Medical Oncology, Department of Biotechnological and Applied Clinical Sciences, University of L'Aquila, L’Aquila, Italy; ^19^ Medical Oncology Unit AOOR Papardo-Piemonte, Department of Human Pathology of Adult And Evolutive Age “Gaetano Barresi”, University of Messina, Messina, Italy; ^20^ Department of Surgical, Oncological and Oral Sciences, Section of Medical Oncology, University of Palermo, Palermo, Italy; ^21^ Medical Oncology, ASST Bergamo Ovest, Ospedale di Treviglio, Bergamo, Italy; ^22^ Medical Oncology, Ospedale F. Renzetti, Lanciano, Italy; ^23^ Department of Pathology, Surgery and Oncology, “Mater Salutis” Hospital, ULSS21, Verona, Italy; ^24^ Medical Oncology, A.O. Ospedale di Insegnamento S. Croce e Carle, Cuneo, Italy; ^25^ Department of Radiotherapy, IRCCS Regina Elena National Cancer Institute, Rome, Italy; ^26^ Medical Oncology, S.M. Goretti Hospital, Latina, Italy; ^27^ Scientific Direction, IRCCS Regina Elena National Cancer Institute, Rome, Italy; ^28^ Institute of General Pathology, Catholic University of the Sacred Heart, Rome, Italy

**Keywords:** metastatic breast cancer, HER2 positive, T-DM1, previous pertuzumab, real-world

## Abstract

We addressed trastuzumab emtansine (T-DM1) efficacy in HER2+ metastatic breast cancer patients treated in real-world practice, and its activity in pertuzumab-pretreated patients. We conducted a retrospective, observational study involving 23 cancer centres, and 250 patients. Survival data were analyzed by Kaplan Meier curves and log rank test. Factors testing significant in univariate analysis were tested in multivariate models. Median follow-up was 15 months and median T-DM1 treatment-length 4 months. Response rate was 41.6%, clinical benefit 60.9%. Median progression-free and median overall survival were 6 and 20 months, respectively. Overall, no differences emerged by pertuzumab pretreatment, with median progression-free and median overall survival of 4 and 17 months in pertuzumab-pretreated (p=0.13), and 6 and 22 months in pertuzumab-naïve patients (p=0.27). Patients who received second-line T-DM1 had median progression-free and median overall survival of 3 and 12 months (p=0.0001) if pertuzumab-pretreated, and 8 and 26 months if pertuzumab-naïve (p=0.06). In contrast, in third-line and beyond, median progression-free and median overall survival were 16 and 18 months in pertuzumab-pretreated (p=0.05) and 6 and 17 months in pertuzumab-naïve patients (p=0.30). In multivariate analysis, lower ECOG performance status was associated with progression-free survival benefit (p<0.0001), while overall survival was positively affected by lower ECOG PS (p<0.0001), absence of brain metastases (p 0.05), and clinical benefit (p<0.0001). Our results are comparable with those from randomized trials. Further studies are warranted to confirm and interpret our data on apparently lower T-DM1 efficacy when given as second-line treatment after pertuzumab, and on the optimal sequence order.

## INTRODUCTION

HER2 is overexpressed/amplified in about 15-20% of breast cancers, and is related to poor prognosis [1, 2]. Trastuzumab has dramatically changed the outcome of these patients, both in the early and advanced setting [3–5]. Unfortunately, all metastatic patients will ultimately develop resistance [6]. Further HER2 blocking agents, such as lapatinib combined with capecitabine, showed activity in trastuzumab pretreated patients [7], and the combination trastuzumab-lapatinib was associated with improved overall survival (OS) compared with lapatinib alone [8]. Trastuzumab emtansine (T-DM1) is a HER2-targeted antibody-drug conjugate comprising DM1, an antimicrotubule maytansine derivative, conjugated to trastuzumab via a stable thioether linker [9]. T-DM1 efficacy in trastuzumab-resistant patients was confirmed in phase II-III trials [10–14] and, in the EMILIA trial, it significantly prolonged progression-free survival (PFS) and OS over lapatinib-capecitabine [12]. In the TH3RESA trial, PFS and OS were significantly improved by T-DM1 over treatment of physician's choice in heavily pretreated metastatic breast cancer (MBC) patients [13, 14], making T-DM1 the mainstay of treatment in patients previously treated with taxane and trastuzumab.

Pertuzumab is a humanized monoclonal antibody binding HER2 at a site distinct from that of trastuzumab and inhibiting HER2 heterodimerization [15]. In the CLEOPATRA trial, pertuzumab, combined with trastuzumab and docetaxel, showed longer PFS and OS over trastuzumab-docetaxel as first-line treatment, being actually the established first-line treatment in HER2 positive (HER2+) MBC [16, 17]. Unfortunately, evidence on T-DM1 efficacy following pertuzumab-trastuzumab-docetaxel is still limited, since available data are mostly from heavily pretreated, pertuzumab-naïve patients. Recently, a retrospective study evaluated T-DM1 activity in 78 pertuzumab-pretreated patients: data showed lower response rate (RR) than usually reported in trastuzumab-resistant patients, even if T-DM1 was delivered for more than 6 months in one third of the patients [18].

We herein present the results from a multicenter, observational study carried out according to a retrospective design. In this trial, we aimed at testing T-DM1 efficacy in a non selected cancer patients population of HER2+ MBC to yield evidence in support of its use in real-world practice.

## RESULTS

We retrospectively identified 250 HER2+ MBC patients treated with T-DM1 from February 2013 through July 2016 at 23 Italian cancer centers. Main patient and tumor characteristics are reported in Table 1. Median age was 56 years, median ECOG PS 0. Sixty-seven patients (26.8%) were metastatic at cancer diagnosis. One hundred and seventy-eight patients had “Luminal B” tumors expressing both (50.4%) or one hormonal receptor/s (20.8%), whereas 72 (28.8%) showed “HER2-enriched” tumors i.e., both ER/PgR negative cancers. Ninety-six patients (38.4%) had received trastuzumab-based regimens in the early setting (neoadjuvant and/or adjuvant). Among the remaining 154 (61.6%) patients, 67 were metastatic at diagnosis, 40 had been treated before trastuzumab registration, and 47 were HER2- at diagnosis. All but 13 patients (5.2%) had been previously treated with one or more HER2-targeted therapies for advanced disease, including trastuzumab/chemotherapy and/or endocrine therapy (ET), lapatinib-capecitabine, pertuzumab-trastuzumab-taxane. Forty-seven (18.8%) patients were pretreated with pertuzumab-based regimens. The median number of previous chemotherapy lines for advanced disease was 2 (0-8). The median number of previous ET lines with/without trastuzumab for advanced disease was 1 (0-4). At T-DM1 starting, 59.2% of the patients had visceral metastases, 24.4% showed asymptomatic brain metastases, 4.4% showed exclusively bone involvement, and 73.2% had multiple metastatic sites. Thirteen patients (5.2%) received T-DM1 as first-line treatment due to recurrence while on or within 6 months from adjuvant treatment, 100 (40%) patients received T-DM1 as second-line, 137 (54.8%) patients were treated in more advanced lines.

**Table 1:** Main baseline characteristics of the study population (number: 250)

Characteristics	Patients, number (%)
AgeMedian (range)	56 (29-82)
HistologyDuctalLobularOther	218 (87.2)15 (6)17 (6.8)
Metastatic at diagnosisYesNo	67 (26.8)183 (73.2)
Grading1-23Unknown	74 (29.6)160 (64)16 (6.4)
HER2-positive at initial diagnosisYesNo	203 (81.2)47 (18.8)
Molecular subtypeTriple-positiveER or PgR positiveHER2-enriched	126 (50.4)52 (20.8)72 (28.8)
ECOG Performance status012	140 (56)91 (36.4)19 (7.6)
Neo-adjuvant chemotherapyYesNot	60 (24)190 (76)
Neoadjuvant/adjuvant trastuzumabYesNot	96 (38.4)154 (61.6)
Adjuvant chemotherapyYesNo	144 (57.6)106 (42.4)
Prior pertuzumab-trastuzumab-taxane treatmentYesNot	47 (18.8)203 (82.2)
T-DM1 administered asFirst-lineSecond-lineThird-line and beyond	13 (5.2)100 (40.0)137 (54.8)
Metastatic sitesVisceralBrainOther	148 (59.2)61 (24.4)41 (16.4)
Number of metastatic sites12≥3	67 (26.8)142 (56.8)41 (16.4)

All but 5 patients were evaluable for efficacy. Among them, 3 refused treatment, while 2 were lost to follow up. Median (m) follow up was 15 months (95%CI, 13-16), and m T-DM1 treatment duration was 4 months (range, 1-29), with 20% of patients having being treated for more than 6 months. Among the 245 evaluable patients, 14 (5.7%) had a complete response (CR) and 95 (38.8%) a partial response (PR), for an overall RR of 44.5% (95%CI, 38.3-50.7). Stable disease (SD) was recorded in 59 patients (24.1%). Clinical benefit (CB), i.e., response or SD lasting ≥6 months, was observed in 145 (59.2%) patients (95%CI, 53.0-65.3). Objective responses and CB by molecular subtype did not differ significantly (Supplementary Table 1). Among the 96 patients who received neoadjuvant/adjuvant trastuzumab, 4 (4.2%) had a CR and 44 (45.8%) a PR, for an overall RR of 50.0% (95%CI, 40-60). Stable disease was recorded in 23 patients (24.0%). Clinical benefit was observed in 61 (63.5%) patients (95%CI, 53.9-73.2). Overall, no differences in responses emerged by disease site (viscera 41%, bone 39%, soft tissue 42%; p=0.95) or by T-DM1 treatment-line, since they were 46.2%, 50%, and 39.6% in first, second, third-line and beyond, respectively (p=0.28).

**Figure 1:**
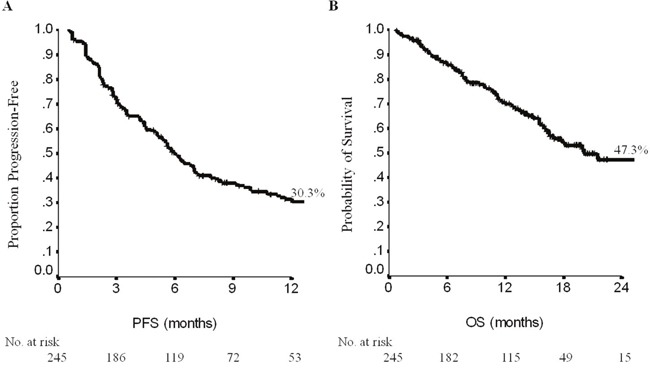
Progression-free survival (PFS, **A**) and overall survival (OS, **B**) in the overall population. No: number.

In the overall patient population, mPFS and mOS were 6 (95%CI, 5-7) and 20 months (95%CI, 14-26), respectively (Figure 1), with no differences by molecular subtype, being mPFS 5.8 months (range, 4.9-6.8) in “Luminal B”, and 7 months (range, 4.7-9.2) in “HER2 enriched” tumours (p=0.29) (Supplementary Table 2). Median OS was 17.8 (range, 13.9-21.8) in “Luminal B”, and 26 months (range, 16-36) in “HER2 enriched” cancers (p=0.14) (Supplementary Table 2 and Supplementary Figure 1). In patients treated with neoadjuvant/adjuvant trastuzumab, median PFS and median OS were 7 (95%CI, 5-9) and 26 months (95%CI, 17-34). Median PFS and OS by metastatic site were 7 (95%CI, 5-9) and 20 months (95%CI, 14-27) in patients without visceral involvement, and 5 (95%CI, 4-6) and 22 months (95%CI, 8-35) in patients with visceral metastases (p=0.07 and p=0.69, respectively). Progression-free survival by T-DM1 treatment-line ranged from 3 to 11 months, with patients treated in third-line showing the most favorable outcome. Median OS was 20 months (95%CI, 13-27) as first-line, 26 months (95%CI, 15.6-36.3) in second-line, and 17.8 months (95%CI, 14-29) when T-DM1 was administered in more advanced lines (Table 2) (p=0.60).

**Table 2:** T-DM1 progression free survival (PFS) and overall survival (OS) according to treatment line

T-DM1 treatment line	mPFS (months)	95%CI	mOS(months)	95%CI	N of pts
**I**	7	6-8	20	13-27	13
**II**	6	5-7	26	15.6-36.3	98
**III**	11	6-16	18	13-23	65
**IV**	6	4-8	18	8-28	38
**V and beyond**	3	2.7-3.3	16	10-21	31

Abbreviations: 95%CI: confidence interval; m: median; N: number; pts: patients.

**Figure 2:**
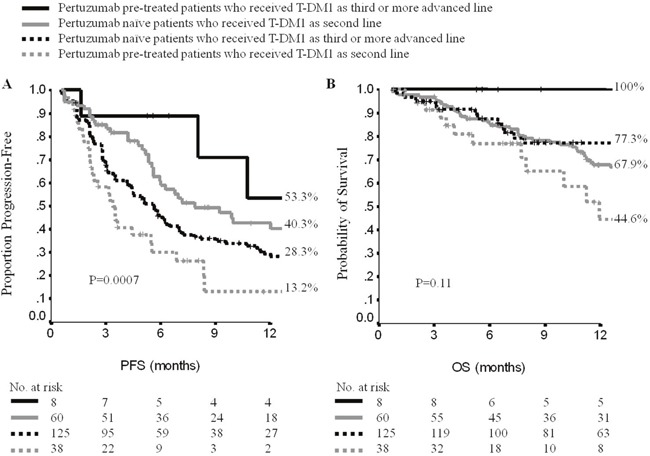
Progression-free survival **(A)** and overall survival **(B)** in patients who received T-DM1 as second-line or beyond according to pertuzumab pre-treatment. PFS: progression-free survival; OS: overall survival; No: number.

An exploratory subgroup analysis was performed in pertuzumab-pretreated patients (47 over 250, Table 3). Overall, RR and CB obtained from T-DM1 in pertuzumab-pretreated patients were 40.2% (95%CI, 26.4-54.4), and 48.9% (95%CI,34.6-63.2), respectively. In pertuzumab-naïve patients, RR and CB were 44.3% (95%CI, 37.5-51.2) and 60.1% (95%CI, 55.4-66.8). Differences were not significant (p=0.75 and p=0.22). Patients who achieved a response/stabilization to previous pertuzumab tended to replicate response/stabilization under T-DM1, even if at a not significant extent (p=0.72, Supplementary Table 3) and independently on T-DM1 line administration. Overall, mPFS and mOS to T-DM1 in pertuzumab-pretreated patients were 4 (95%CI, 2-7) and 17 months (95%C.I., 11-22), respectively In pertuzumab-naïve patients, mPFS was 6 months (95%CI, 5-7), and mOS was 22 months (95%CI, 14-29). These differences were not significant (p=0.13 and 0.27, respectively). We further analyzed the impact of pertuzumab-pretreatment by line of T-DM1 administration. Pertuzumab-pretreated patients who received T-DM1 as second-line (39 patients), showed a mPFS of 3 months (95%CI, 2-4), whereas 62 patients, who did not receive previous pertuzumab but other antiHER2-based treatments and received second-line T-DM1, had a mPFS of 8 months (95%CI, 4-12) (p=0.0001) (Figure 2, Supplementary Table 4). Results were confirmed when adjusting for propensity score (Supplementary Figure 2). This minimizes the chances that the differences observed between the groups compared were driven by unevenly distributed baseline characteristics for the patients included. These latter characteristics are shown in Supplementary Table 5. Median OS for pertuzumab-pretreated patients who received second-line T-DM1 was 12 months (95%CI, 9-15), whereas it was 26 months (95%CI, 16-36) in pertuzumab-naïve patients (p=0.06) (Figure 2). The small subset of 8 patients who received prior pertuzumab and were treated with T-DM1 as third-line and beyond, showed a longer mPFS compared with those treated with second-line T-DM1 following pertuzumab/trastuzumab (16 *vs* 3 months, p=0.004). In more details, 4 patients progressed on T-DM1 at 3, 8, 11 and 16 months. The remaining 4 patients were progression-free at 6 (2 patients), 15 (1 patient) and 24 months (1 patient). Moreover, patients receiving second-line T-DM1 after pertuzumab, showed a significantly shorter mOS than that reported in pertuzumab-pretreated patients receiving T-DM1 beyond the second-line (12 *vs* 18 months, p=0.04). One-hundred and twenty-five pertuzumab-naïve patients receiving T-DM1 beyond the second-line had a mPFS of 6 months (95%CI, 4-7), and a mOS of 17 months (95%CI, 12-22), with no relevant statistical differences (p=0.05 and p 0.30, respectively) (Figure 2, Supplementary Table 4).

**Table 3:** T-DM1 responses according to pertuzumab-pretreatment

	Pertuzumab pre-treated patients (95%CI)	Pertuzumab naïve patients (95%CI)	p
**Response rate**	40.2% (26.4-54.4)	44.3% (37.5-51.2)	0.75
**Clinical benefit rate**	48.9% (34.6-63.2)	60.1% (55.4-66.8)	0.22

Abbreviations: 95%CI: confidence interval.

In multivariate analysis including the overall population (Table 4), the only factor related to PFS benefit was lower ECOG PS (p<0.0001), while OS was positively affected by lower ECOG PS (p=0.001), CB (p<0.0001), and absence of brain metastases at baseline (p=0.05).

**Table 4:** Multivariate analysis

PFS	HR	95%CI	P
ECOG PS(1-2 vs 0)	2.21	1.64-2.98	<0.0001
**OS**	**HR**	**95%CI**	**P**
ECOG PS(1-2 vs 0)	2.28	1.41-3.68	0.001
Brain metastases(yes vs no)	1.60	1-2.57	0.05
CB(no vs yes)	3.83	2.42-6.06	<0.0001

Abbreviations: HR: hazard ratio; 95%CI: confidence interval; PS: performance status; CB: clinical benefit.

Overall, disease progression involved the central nervous system (CNS) in 44 (17.6%) patients, 11.6% in patients without previous brain metastases, 36.1% in patients with previous CNS involvement. Patients with CNS metastases at baseline (61 patients) showed similar mPFS compared to the overall population (6 months, 95%CI, 4-8), while mOS was shorter (16 months, 95%CI, 12-19).

Data on toxicity were only partially available due to the observational and retrospective design, and are showed in Supplementary Table 6. Mild and transient cardiac dysfunction was observed in 4 patients (1.6%).

## DISCUSSION

We analyzed data from 250 HER2+ MBC patients treated with T-DM1 at 23 Italian cancer centers. On the whole, our results on T-DM1 activity expressed in terms of RR and CB favorably compare with those from phase II-III trials [11–13]. Overall, our data seem not to provide support to differences in T-DM1 efficacy by prior exposure to pertuzumab. However, generally, patients with more favorable outcomes to pertuzumab tended to show favorable outcomes also to T-DM1, although at a not statistically significant extent. When stratifying analysis by line of T-DM1 administration, pertuzumab-pretreated patients who received T-DM1 in second-line showed worse survival outcomes compared to pertuzumab-naïve patients. Conversely, when T-DM1 was administered in more advanced lines, mPFS was slightly better in pertuzumab-pretreated patients. Interestingly, no significant differences emerged when patients having received T-DM1 in second *vs* more advanced lines were compared by mPFS following exposure to pertuzumab. This may suggest that patients from the subsets compared do not necessarily represent two distinct populations with different sensitivity to HER2 blocking agents.

Based on our data, T-DM1 efficacy seems independent on the line of treatment in pertuzumab-naïve patients, whereas, in pertuzumab-pretreated patients, T-DM1 showed greater efficacy when not administered immediately after pertuzumab-based treatment. Several keys of interpretation may be provided in an attempt to clarify our findings. However, the restricted number of patients invites extreme caution in interpreting our results, since pertuzumab-pretreated patients were only 47, and patients treated with T-DM1 beyond the second-line only 8. In addition, the use of pertuzumab within a 3-drug combination including trastuzumab and a taxane may add a substantial degree of complexity to the interpretation of our findings. We may hypothesize that patients who received T-DM1 following multiple HER2 blocking-based treatments had an intrinsically less aggressive disease that allowed a higher number of therapeutic lines. This hypothesis may be somewhat supported by the results of correlation analysis between the number of lines of treatment and survival outcomes in subgroups defined upon previous exposure to pertuzumab. In brief, in the subgroup of patients previously exposed to pertuzumab, we observed a direct correlation between the number of previous lines of treatment and both mPFS (R = 0.50, p = 0.003) and mOS (R=0,61, p=0.02). When considering patients naïve to pertuzumab, the number of lines of treatment and mPFS were inversely related (R = -0.23, p=0.005), while no relevant associations emerged with OS (p=0.94) (data available upon request).

It is also plausible that when T-DM1 administration immediately follows pertuzumab-based combinations because of resistance occurrence, its activity may suffer from a transient impaired access of this drug to the binding site. This latter may be hypothesized based on the lack of HER2 receptors on tumor cells surface due to pertuzumab-trastuzumab-mediated internalization with endocytic destruction, or to inefficient prevention of proteolitic shedding of extracellular domain, resulting in truncated forms (p95HER2) [19–23]. Conversely, the 8 pertuzumab-pretreated patients who received other regimens prior to T-DM1, (such as lapatinib-based combination, or chemotherapy plus trastuzumab), could have benefited the most from subsequent T-DM1 because of the re-expression of HER2 receptors on tumor cells surface, partially related to lapatinib [24, 25], or simply due to the time-interval elapsed between pertuzumab and T-DM1 administration. Moreover, a chemotherapeutic agent not directly conjugated to trastuzumab but given in combination with it, in patients with a transient limited access to HER2 receptors, may be efficacious even if administered immediately after pertuzumab/trastuzumab. Conversely, the activity of T-DM1 is strictly related to the presence of surface HER2 receptors, since after T-DM1 binds to HER2 receptors, the HER2-T-DM1 complex enters into cancer cells through receptor-mediated endocytosis, and releases DM1. The limits imposed by the restricted number of patients in our subgroup analysis, along with the retrospective nature of our study design, call for confirmation of our findings in future, prospectively designed and opportunely sized studies.

Because pertuzumab and T-DM1 have been recently approved for first and second-line treatment of HER2 MBC, several patients have not been treated with these agents yet, making it difficult a truly comprehensive evaluation of the efficacy of the optimal sequence order. A recent report on 78 pertuzumab-pretreated patients treated with T-DM1 in routine practice as first, second and more advanced lines [18] showed lower rates of responses compared to previous studies of trastuzumab-resistant patients, even if 30.8% of patients received T-DM1 for at least 6 months. Twenty-six patients were treated as first or second-line and 52 as more advanced lines. When T-DM1 was administered as first or second-line, the authors reported a clinical response of 23.1%, and a prolonged duration on therapy in 34.6% of patients. In more advanced lines, T-DM1 showed a clinical response of 15.4% and a prolonged duration on therapy in 28.8% of patients. In patients pretreated with lapatinib the response was 11.1% and T-DM1 was administered for more than 6 months in 27.8% of patients. There are some differences in the patients’ populations from these two studies. Primarily, the percentage of patients presenting with *de novo* stage IV disease (44% in the Dzimitrowitz study and 27% in the present study); moreover, our patients exhibited more often hormonal receptor positive tumors (71.2% versus 62.2%). In addition, in the study from Dzimitrowitz, when reporting results on T-DM1 activity, no distinction is made between first- or second-line of administration. Another recent report from the T-PAS expanded access study of T-DM1 in heavily pretreated patients, showed a mT-DM1 duration of 5.0 months and a RR of 25.6%, with a safety profile comparable with that of phase II-III studies [26]. However, these patients were all pertuzumab-naïve.

At present, few data are available regarding efficacy of T-DM1 in breast cancer patients with CNS metastases. In our study 22 patients developed CNS as first-site of progression (11.6%), whereas twenty-two (36.1%) patients with known and pretreated CNS metastases developed progression at CNS site. Overall, mPFS in patients with CNS metastases at baseline was similar to that of patients without CNS metastases; conversely, mOS was shorter. In a multicenter retrospective study of T-DM1 administered to patients with known CNS metastases, this drug activity was confirmed, with results comparable to those of patients without brain metastases, except for OS [27]. Conversely, in a retrospective analysis of the EMILIA trial in patients with known CNS metastases, a significant improvement in OS was observed in the T-DM1 arm versus lapatinib-capecitabine, whereas PFS, and the rate of progression at CNS site, were similar in the two arms [28]. A more exhaustive analysis on T-DM1 activity in our patient population with known or developing brain metastases is ongoing, and will be soon reported in a separate manuscript.

Toxicity data from our patient population were only partly available, due to retrospective study design applied to the real world setting. Overall, treatment with T-DM1 was generally well tolerated. No grade 4 toxicities were recorded, and grade 3 adverse events were uncommon, mostly fatigue, thrombocytopenia, increase in serum transaminases and nausea, with no new safety issue.

The present study has some important limitations, mostly related to its retrospective design, and to the heterogeneity of the study population. Indeed, the fairly high number of participating centers and the real-world practice setting certainly concurred to add heterogeneity to our study population compared to patients from randomized clinical studies. Moreover, in retrospective studies, the RECIST criteria and timing at tumor re-assessment are less stringent and precise than in prospective trials. In addition, although our study population may seem not particularly limited in size, caution must be paid when interpreting results from our subgroup analyses. Indeed, the limited number of patients included in subsets defined upon the variables of interest may importantly limit the statistical power of some of the analyses performed and the generalizability of the results obtained.

When compared by prior exposure to pertuzumab, patients differed significantly in terms of age (Supplementary Table 5). Pertuzumab-naïve patients also showed a lower rate of visceral metastases, although at a not significant extent. To minimize the above reported selection bias, survival analyses were adjusted by propensity score. However, case matching by age and visceral metastases could not remove other potentially important causes of bias from unknown confounders possibly including prior/subsequent therapies, comorbidities, and differences in disease biology. In these regards, it is worth mentioning that data concerning co-morbidities and safety were not available for analysis purposes. Furthermore, the lack of punctual details concerning drug exposure in terms of changes eventually occurred in the administration schedule may have somewhat limited our ability to correctly interpret our study results. More generally, missing data on variables and outcomes of possible interest, i.e., toxicity, derive from our limited ability to gather all the relevant information for depicting a complete patient profile while working in a real world setting, particularly when relying on a retrospective approach.

Our study also has some relevant strengths. First, it reports outcomes of T-DM1 treatment in a large cohort of HER2+ MBC patients treated outside clinical trials in the real-world setting, thus coherently reproducing the daily practice. Moreover, to our knowledge, this is the first study on T-DM1 activity reporting results in light of pertuzumab pretreatment and lines of T-DM1 administration. Even if numbers are relatively small and results are not from a randomized prospectively designed study, our report on the apparently lower efficacy of T-DM1 when this latter is administered right after pertuzumab-trastuzumab deserves further investigations. Since HER2+ breast cancer evolves under selective pressure of new targeted agents, it is of paramount importance recognizing unexpected resistance pathways, partially related also to novel treatments sequence order.

At present, the definition of the right sequence of HER2 blocking agents remains a challenge. While waiting for the results of the ongoing prospective trial testing T-DM1 in pertuzumab-pretreated patients (NCT01835236), our study may offer some interesting clues on a non-selected HER2+ MBC population treated with T-DM1 after multiple HER2 blocking agents-based therapies.

## PATIENTS AND METHODS

We retrospectively identified patients who received T-DM1 at various Italian oncologic centres. The follow-up was stopped in August 2016, that is, when a median follow up of at least 12 months was reached and statistical analysis performed. Our primary objective was evaluating T-DM1 efficacy in a non-selected patient population. Secondarily, we assessed its activity in pertuzumab-pretreated patients. T-DM1 was administered according to guidelines until disease progression, unacceptable toxicity, or patient refusal. Treatment efficacy was evaluated by conventional RECIST criteria. Toxicity data, when available, were graded using the NCI-CTCAE (version 4.0). Our study was approved by local Ethic Committees and conducted according to the Helsinki Declaration. All the patients released a written informed consent.

### Data collection

Medical records were retrieved for demographic, clinical and molecular features, previous treatments and related outcomes, number and site of metastases at the time of T-DM1 starting, tumor response, toxicity, date at disease progression, date at the last follow-up or death. Pathology assessment was performed in surgical specimens of primary tumors at the participating centers. When missing, the molecular features were centrally evaluated in formalin-fixed, paraffin-embedded tissue sections. Anonymized data were entered into a dedicated database.

### Statistical analysis

Variables were assessed by Pearson Chi-Square test or Fisher Exact test. Their impact on survival was tested in Cox uni/multivariate models. Significance was set at *p*≤0.05. The multivariate Cox hazard model was built using stepwise regression (forward selection). Enter and remove limit were *p*=0.10 and *p*=0.15. The following variables were considered: age, ECOG PS, histology, ki67, molecular subtype, stage at diagnosis, type of surgery, adjuvant and number of advanced treatments, disease-free survival, pertuzumab pretreatment, type and number of metastatic sites and treatment response. Survival was addressed by the Kaplan–Meier method and log-rank test. Significance was defined at *p*≤0.05 level.

The effect of covariates potentially acting as confounders in a non-randomized cohort was minimized by propensity score match, which allowed to create patient groups who were similarly likely to receive a given treatment based on their baseline characteristics [29]. SPSS software was used for statistical evaluations (SPSS version 21.0, SPSS Inc., Chicago, Illinois, USA).

## SUPPLEMENTARY MATERIALS FIGURES AND TABLES



## Abbreviations

MBC: metastatic breast cancer
CB: clinical benefit
mPFS: median progression free survival
mOS: median overall survival
OS: overall survival
T-DM1: trastuzumab emtansine
PFS: progression-free survival
HER2+: HER2 positive
RR: response rate
ET: endocrine therapy
M: median
CR: complete response
PR: partial response
SD: stable disease
CB: clinical benefit
CNS: central nervous system
95%CI: confidence interval
HR: hazard ratio
PS: performance status
No: number

## References

[1] Dawood S, Broglio K, Buzdar AU, Hortobagyi GN, Giordano SH (2010). Prognosis of women with metastatic breast cancer by HER2 status and trastuzumab treatment: an institutional-based review. J Clin Oncol.

[2] Giordano SH, Temin S, Kirshner JJ, Chandarlapaty S, Crews JR, Davidson NE, Esteva FJ, Gonzalez-Angulo AM, Krop I, Levinson J, Lin NU, Modi S, Patt DA (2014). Systemic therapy for patients with advanced human epidermal growth factor receptor 2-positive breast cancer: American Society of Clinical Oncology clinical practice guideline. J Clin Oncol.

[3] Viani GA, Afonso SL, Stefano EJ, De Fendi LI, Soares FV (2007). Adjuvant trastuzumab in the treatment of HER-2-positive early breast cancer: a meta-analysis of published randomized trials. BMC Cancer.

[4] Slamon DJ, Leyland-Jones B, Shak S, Fuchs H, Paton V, Bajamonde A, Fleming T, Eiermann W, Wolter J, Pegram M, Baselga J, Norton L (2001). Use of chemotherapy plus a monoclonal antibody against HER2 for metastatic breast cancer that overexpresses HER2. N Engl J Med.

[5] Marty M, Cognetti F, Maraninchi D, Snyder R, Mauriac L, Tubiana-Hulin M, Chan S, Grimes D, Antón A, Lluch A, Kennedy J, O’Byrne K, Conte P (2005). Randomized phase II trial of the efficacy and safety of trastuzumab combined with docetaxel in patients with human epidermal growth factor receptor 2-positive metastatic breast cancer administered as first-line treatment: the M77001 study group. J Clin Oncol.

[6] Recondo G, de la Vega M, Galanternik F, Díaz-Cantón E, Leone BA, Leone JP (2016). Novel approaches to target HER2-positive breast cancer: trastuzumab emtansine. Cancer Manag Res.

[7] Geyer CE, Forster J, Lindquist D, Chan S, Romieu CG, Pienkowski T, Jagiello-Gruszfeld A, Crown J, Chan A, Kaufman B, Skarlos D, Campone M, Davidson N (2006). Lapatinib plus capecitabine for HER2-positive advanced breast cancer. N Engl J Med.

[8] Blackwell KL, Burstein HJ, Storniolo AM, Rugo HS, Sledge G, Aktan G, Ellis C, Florance A, Vukelja S, Bischoff J, Baselga J, O’Shaughnessy J (2012). Overall survival benefit with lapatinib in combination with trastuzumab for patients with human epidermal growth factor receptor 2-positive metastatic breast cancer: final results from the EGF104900 Study. J Clin Oncol.

[9] Lewis Phillips GD, Li G, Dugger DL, Crocker LM, Parsons KL, Mai E, Blättler WA, Lambert JM, Chari RV, Lutz RJ, Wong WL, Jacobson FS, Koeppen H (2008). Targeting HER2-positive breast cancer with trastuzumab-DM1, an antibody-cytotoxic drug conjugate. Cancer Res.

[10] Burris HA, Rugo HS, Vukelja SJ, Vogel CL, Borson RA, Limentani S, Tan-Chiu E, Krop IE, Michaelson RA, Girish S, Amler L, Zheng M, Chu YW (2011). Phase II study of the antibody drug conjugate trastuzumab-DM1 for the treatment of human epidermal growth factor receptor 2 (HER2)-positive breast cancer after prior HER2-directed therapy. J Clin Oncol.

[11] Krop IE, LoRusso P, Miller KD, Modi S, Yardley D, Rodriguez G, Guardino E, Lu M, Zheng M, Girish S, Amler L, Winer EP, Rugo HS (2012). A phase II study of trastuzumab emtansine in patients with human epidermal growth factor receptor 2-positive metastatic breast cancer who were previously treated with trastuzumab, lapatinib, an anthracycline, a taxane, and capecitabine. J Clin Oncol.

[12] Verma S, Miles D, Gianni L, Krop IE, Welslau M, Baselga J, Pegram M, Oh DY, Diéras V, Guardino E, Fang L, Lu MW, Olsen S (2012). Trastuzumab emtansine for HER2-positive advanced breast cancer. N Engl J Med.

[13] Krop IE, Kim SB, González-Martín A, LoRusso PM, Ferrero JM, Smitt M, Yu R, Leung AC, Wildiers H, TH3RESA Study Collaborators (2014). Trastuzumab emtansine versus treatment of physician’s choice for pretreated HER2-positive advanced breast cancer (TH3RESA): a randomised, open-label, phase 3 trial. Lancet Oncol.

[14] Wildiers H, Kim SB, Gonzalez-Martin A, LoRusso PM, Ferrero M, Yu R, Smitt M, Krop I (2015). Trastuzumab emtansine improves overall survival versus treatment of physician’s choice in patients with previously treated HER2-positive metastatic breast cancer: final overall survival results from the phase 3 TH3RESA study. SABCS Meeting Abstract.

[15] Franklin MC, Carey KD, Vajdos FF, Leahy DJ, de Vos AM, Sliwkowski MX (2004). Insights into ErbB signaling from the structure of the ErbB2-pertuzumab complex. Cancer Cell.

[16] Baselga J, Cortés J, Kim SB, Im SA, Hegg R, Im YH, Roman L, Pedrini JL, Pienkowski T, Knott A, Clark E, Benyunes MC, Ross G (2012). Pertuzumab plus trastuzumab plus docetaxel for metastatic breast cancer. N Engl J Med.

[17] Swain SM, Kim SB, Cortés J, Ro J, Semiglazov V, Campone M, Ciruelos E, Ferrero JM, Schneeweiss A, Heeson S, Clark E, Ross G, Benyunes MC (2013). Pertuzumab, trastuzumab, and docetaxel for HER2-positive metastatic breast cancer (CLEOPATRA study): overall survival results from a randomised, double-blind, placebo-controlled, phase 3 study. Lancet Oncol.

[18] Dzimitrowicz H, Berger M, Vargo C, Hood A, Abdelghany O, Raghavendra AS, Tripathy D, Valero V, Hatzis C, Pusztai L, Murthy R (2016). T-DM1 activity in metastatic human epidermal growth factor receptor 2-positive breast cancers that received prior therapy with trastuzumab and pertuzumab. J Clin Oncol.

[19] Arribas J, Baselga J, Pedersen K, Parra-Palau JL (2011). p95HER2 and breast cancer. Cancer Res.

[20] Scaltriti M, Rojo F, Ocaña A, Anido J, Guzman M, Cortes J, Di Cosimo S, Matias-Guiu X, Ramon y, Cajal S, Arribas J, Baselga J (2007). Expression of p95HER2, a truncated form of the HER2 receptor, and response to anti-HER2 therapies in breast cancer. J Natl Cancer Inst.

[21] Hudis CA (2007). Trastuzumab--mechanism of action and use in clinical practice. N Engl J Med.

[22] Capelan M, Pugliano L, De Azambuja E, Bozovic I, Saini KS, Sotiriou C, Loi S, Piccart-Gebhart MJ (2013). Pertuzumab: new hope for patients with HER2-positive breast cancer. Ann Oncol.

[23] Molina MA, Codony-Servat J, Albanell J, Rojo F, Arribas J, Baselga J (2001). Trastuzumab (Herceptin), a humanized anti-HER2 receptor monoclonal antibody, inhibits basal and activated HER2 ectodomain cleavage in breast cancer cells. Cancer Res.

[24] Scaltriti M, Verma C, Guzman M, Jimenez J, Parra JL, Pedersen K, Smith DJ, Landolfi S, Ramon y, Cajal S, Arribas J, Baselga J (2009). Lapatinib, a HER2 tyrosine kinase inhibitor, induces stabilization and accumulation of HER2 and potentiates trastuzumab-dependent cell cytotoxicity. Oncogene.

[25] Scaltriti M, Chandarlapaty S, Prudkin L, Aura C, Jimenez J, Angelini PD, Sánchez G, Guzman M, Parra JL, Ellis C, Gagnon R, Koehler M, Gomez H (2010). Clinical benefit of lapatinib-based therapy in patients with human epidermal growth factor receptor 2-positive breast tumors coexpressing the truncated p95HER2 receptor. Clin Cancer Res.

[26] Yardley DA, Krop IE, LoRusso PM, Mayer M, Barnett B, Yoo B, Perez EA (2015). Trastuzumab emtansine (T-DM1) in patients with HER2-positive metastatic breast cancer previously treated with chemotherapy and 2 or more HER2-targeted agents: results from the T-PAS expanded access study. Cancer J.

[27] Jacot W, Pons E, Frenel JS, Guiu S, Levy C, Heudel PE, Bachelot T, D’Hondt V, Darlix A, Firmin N, Romieu G, Thezenas S, Dalenc F (2016). Efficacy and safety of trastuzumab emtansine (T-DM1) in patients with HER2-positive breast cancer with brain metastases. Breast Cancer Res Treat.

[28] Krop IE, Lin NU, Blackwell K, Guardino E, Huober J, Lu M, Miles D, Samant M, Welslau M, Diéras V (2015). Trastuzumab emtansine (T-DM1) versus lapatinib plus capecitabine in patients with HER2-positive metastatic breast cancer and central nervous system metastases: a retrospective, exploratory analysis in EMILIA. Ann Oncol.

[29] D’Agostino RB (1998). Propensity score methods for bias reduction in the comparison of a treatment to a non-randomized control group. Stat Med.

