# Supplementary material for: A retrospective multicentric observational study of trastuzumab emtansine in HER2 positive metastatic breast cancer: a real-world experience
Source: Oncotarget. 2017 May 25;8(34):56921–31. doi: 10.18632/oncotarget.18176 (PMC5593613; doi:10.18632/oncotarget.18176)
Supplement: Supplementary file 1 [file oncotarget-08-56921-s001.pdf]

A retrospective multicentric observational study of trastuzumab emtansine in HER2 positive metastatic breast cancer: a real-world experience

SUPPLEMENTARY MATERIALS

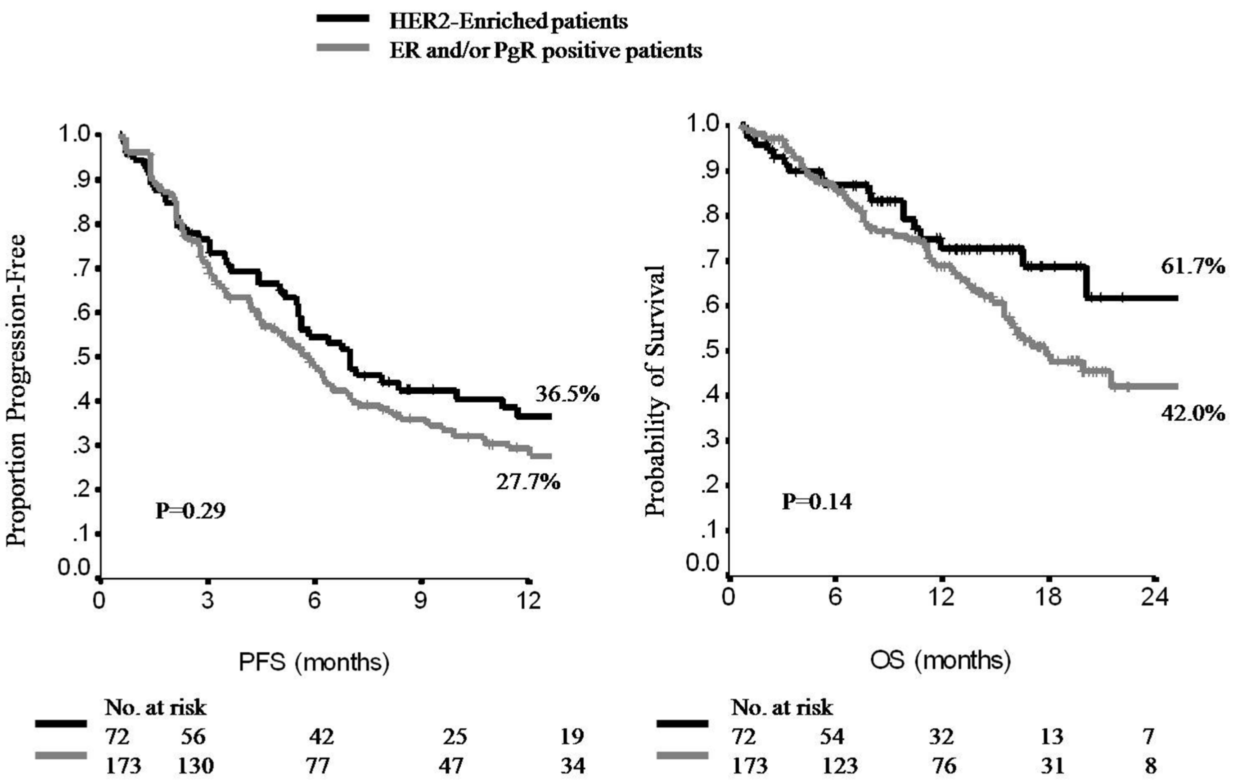

Supplementary Figure 1: Progression-free survival **(A)** and overall survival **(B)** according to molecular subtype. PFS: progression-free survival; OS: overall survival; No: number; ER: estrogen receptor; PgR: progesterone receptor.

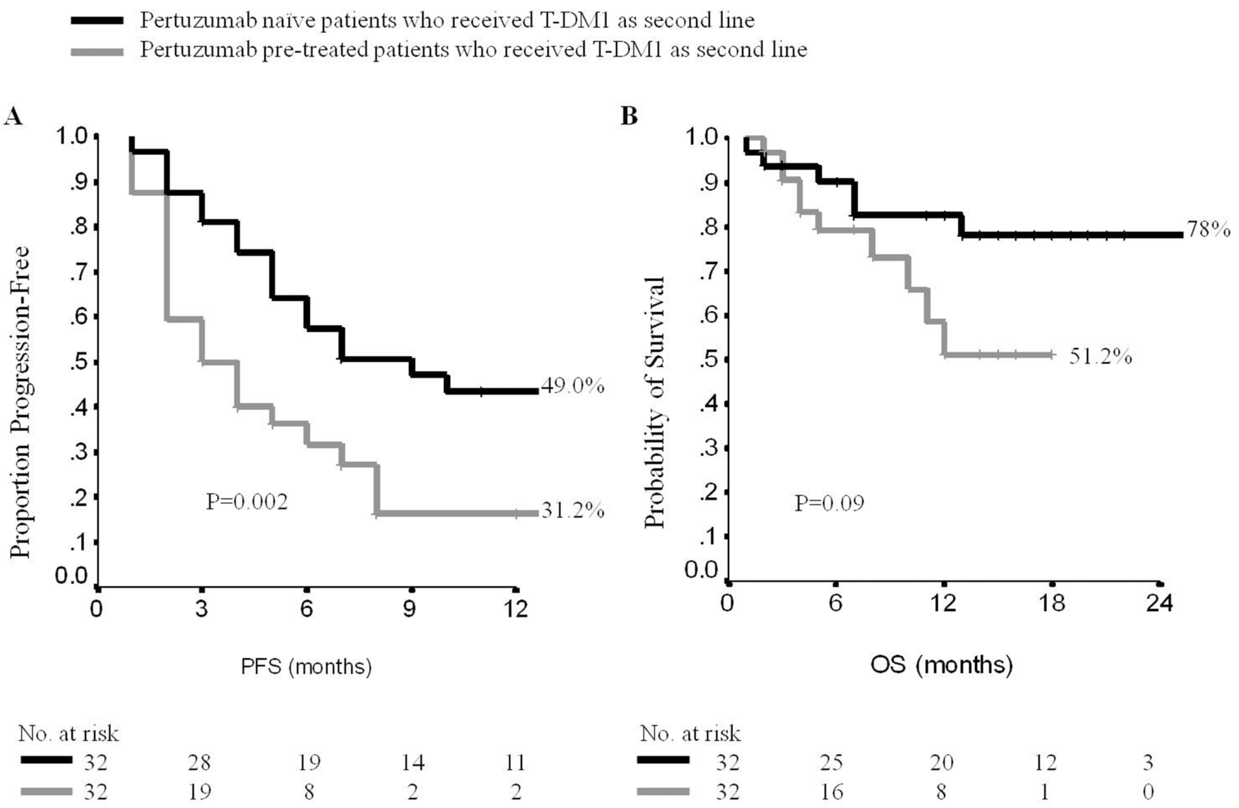

Supplementary Figure 2: Progression-free survival **(A)** and overall survival **(B)** in patients who received T-DM1 as second-line according to pertuzumab pre-treatment adjusted for propensity score. PFS: progression-free survival; OS: overall survival; No: number.

Supplementary Table 1: T-DM1 responses according to molecular subtypes

Response	Overall	Molecular subtype		p
		ER and/or PgR positive	HER2-Enriched	
Complete/partial response	109 (44.5%)	74 (43.0%)	35 (49.0%)	0.16
Stable disease	59 (24.1%)	39 (22.0%)	20 (28.0%)	
Progressive disease	77 (31.4%)	60 (35.0%)	17 (23.0%)	
Clinical benefit rate	145 (59.2%)	97 (56.0%)	48 (67.0%)	0.16

Supplementary Table 2: T-DM1 progression free survival (PFS) and overall survival (OS) according to molecular subtype

Molecular subtype	mPFS (months)	95%CI	p	mOS (months)	95%CI	p
**Luminal B**	5.8	4.9-6.8	0.29	17.8	13.9-21.8	0.14
**HER2-enriched**	7	4.7-9.2		26	16-36	

Abbreviations: 95%CI: confidence interval; m: median.

Supplementary Table 3: T-DM1 responses according to previous pertuzumab responses (46 patients evaluable for both treatments)

Pertuzumab response	T-DM1 response		
	Complete/partial response	Stable disease	Progressive disease
Complete/partial response	14 (45.2%)	6 (19.4%)	11 (35.5%)
Stable disease	4 (36.4%)	3 (27.3%)	4 (36.4%)
Progressive disease	1 (25.0%)	1 (25.0%)	2 (50.0%)
Total	19 (41.3%)	10 (21.7%)	17 (37%)

Supplementary Table 4: Progression-free survival (PFS) and overall survival (OS) in patients who received T-DM1 as second-line or beyond according to pertuzumab pre-treatment

T-DM1 line	Median PFS, months (95%CI)			Median OS, months (95%CI)		
	Pertuzumab pre-treated patients	Pertuzumab naïve patients	*p*	Pertuzumab pre-treated patients	Pertuzumab naïve patients	*p*
Second	3 (2-4)	8 (4-12)	*0.0001*	12 (9-15)	26 (16-36)	*0.06*
Third or beyond	16 (8-23)	6 (4-7)	*0.05*	18 (17-24)	17 (12-22)	*0.3*

Abbreviations: 95%CI: confidence interval.

Supplementary Table 5: Main baseline characteristics of patients who received T-DM1 as second-line according to pertuzumab-pretreatment

Variables	N (101 pts)	Pertuzumab naïve patients (62 pts) n(%)	Pertuzumab pretreated patients (39 pts) n(%)	P
Age				
<56	53	27 (43.5)	26 (66.7)	0.02
>56	48	35 (56.5)	13 (33.3)	
ECOG PS				
0	59	37 (59.7)	22 (56.4)	0.75
1-2	42	25 (40.3)	17 (43.6)	
DFS from diagnosis				
Metastatic at diagnosis	25	14 (22.6)	11 (28.2)	0.63
DFS≤24 months	25	20 (32.3)	5 (12.8)	
DFS>24 months	51	28 (45.2)	23 (59.0)	
Molecular subtype				
Luminal B	63	40 (64.5)	23 (59.0)	0.53
HER2-Enriched	38	22 (35.5)	16 (41.0)	
Histology				
Ductal	90	57 (91.9)	33 (84.6)	0.33
Other	11	5 (8.1)	6 (15.4)	
Visceral metastases				
No	37	26 (41.9)	11 (28.2)	0.16
Yes	64	36 (58.1)	28 (71.8)	
Brain metastases				
No	71	46 (74.2)	25 (64.1)	0.28
Yes	30	16 (25.8)	14 (35.9)	
Number of metastatic sites				
1	44	27 (43.5)	17 (43.6)	0.99
>1	57	35 (56.5)	22 (56.4)	

Abbreviations: N: number; DFS: disease-free survival; PS: performance status.

Supplementary Table 6: Main toxicity in the overall population (N:250) according to National Cancer Institute Common Terminology Criteria version 4.0

Toxicity	Grade 1<br>N(%)	Grade 2<br>N(%)	Grade 3<br>N(%)
Hypertransaminasemia	61 (24.4)	14 (5.6)	1 (0.4)
Fatigue	49 (19.6)	29 (11.6)	5 (2)
Nausea	33 (11.2)	7 (2.8)	2 (0.8)
Thrombocytopenia	26 (10.4)	12 (4.8)	3 (1.2)
Diarrhea	7 (2.8)	2 (0.8)	-

Abbreviations: N: number.
